# Publisher Correction: Low replicability can support robust and efficient science

**DOI:** 10.1038/s41467-020-17924-9

**Published:** 2020-08-11

**Authors:** Stephan Lewandowsky, Klaus Oberauer

**Affiliations:** 1 School of Psychological Science, University of Bristol, 12A, Priory Road, Bristol, BS8 1TU UK; 2 School of Psychological Science, University of Western Australia, Perth, WA Australia; 3 Department of Psychology, University of Zurich, Zurich, Switzerland

**Keywords:** Research management, Psychology

Correction to: *Nature Communications* 10.1038/s41467-019-14203-0, published online 17 January 2020.

The original HTML version of this Article was updated after publication because the previous HTML version linked to an incorrect Source data file. As Source data were not provided in this file, this link has been removed. The original Data availability statement is correct and includes a link where data and analysis code can be accessed.

