# Supplementary material for: Low replicability can support robust and efficient science
Source: Nat Commun. 2020 Jan 17;11:358. doi: 10.1038/s41467-019-14203-0 (PMC6969070; doi:10.1038/s41467-019-14203-0)
Supplement: Supplementary file 1 — Supplementary Information [file 41467_2019_14203_MOESM1_ESM.pdf]

Supplementary Material for: Low replicability can support robust and efficient science

Stephan Lewandowsky
University of Bristol

Klaus Oberauer
University of Zurich

Exploration of thresholds for scientific interest

The simulations in the main article used the 90th percentile of the distribution fitted to citation data ($q = 22.98$ citations) as a threshold in a logistic transfer function to determine the scientific community's interest in a phenommenon:

$$P(I_k) = \frac{1}{1 + \mathrm{e}^{-(n_k - q)/t}},$$

where $P(I_k)$ is the probability that finding k would be deemed interesting, n_k represents the finding's citation count, and $t \in \{1, 5, 10\}$ the temperature of the logistic function. We explored other cutoffs spanning the range from the 10th through the 90th percentile of the citation distribution (in decile steps). Figure S1 shows the results for four cutoffs (10th, 30th, 50th, and 70th percentile) using discovery oriented research with frequentist statistics.

The figure shows that the cost advantage of the private replication regime is independent of the particular threshold being used. The figure also shows that the effect of temperature on the number of experiments and discovered phenomena reverses across settings of the threshold. As illustrated in Figure S2, this occurs because at higher threshold settings (above the median), greater temperature translates into many additional findings with citations below the threshold being replicated (because the logistic has a shallower slope and articles with few citations are more likely to be sampled than articles with higher citation counts). With lower thresholds, the lower half of the logistic function is cut off (at 0 citations), and hence the shallower slope only contributes to a reduction of the number of studies falling above the threshold that are being replicated.

Effects of fraud

In this simulation all experiments during the first round were arbitrarily declared significant irrespective of the actual outcome and irrespective of replication regime. Thus, for the private replication regime, there were always 200 experiments that represented the original 100 studies from the first round, all declared significant, plus their replications.

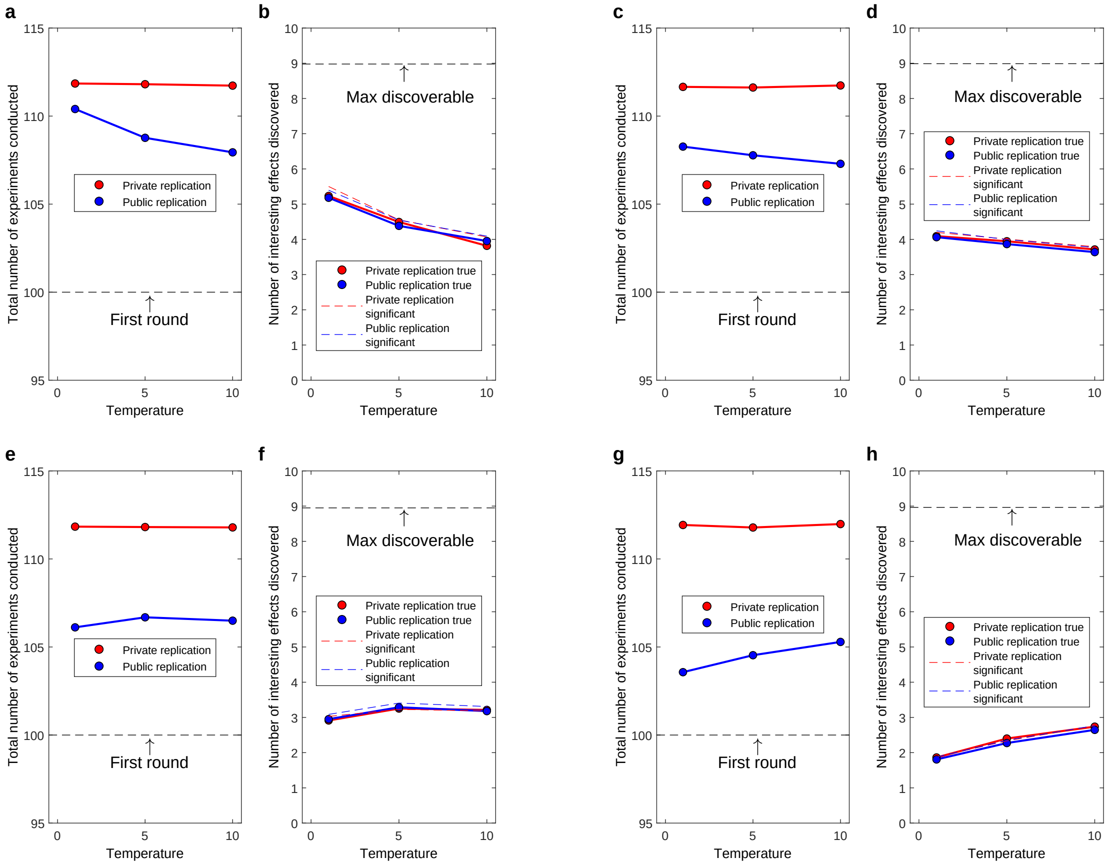

Figure S1. Comparison of different thresholds of interest. Panels **a** and **b** use a threshold at the 10th percentile of the distribution of citations; panels **c** and **d** use a threshold at the 30th percentile; panels **e** and **f** use a threshold at the 50th percentile; and panels **g** and **h** use a threshold at the 70th percentile. All simulations in the main text used a threshold at the 90th percentile.

Public replications, by contrast, were honest but handicapped by the fact that most of them sought a replication of non-existent effects.

Figure S3 illustrates the consequences of fraud. Panel **a** shows the greater cost in terms of experiments conducted by other investigators compared to a non-fraud scenario (Figure 3 in main text). Panel **b** shows that fraud engenders a greater number of falsely-identified interesting effects (reflected in the difference between the dashed and solid lines) because all Type I errors of the honest replications are interpreted as successful replications of (non-existent) studies.

Pursuit of non-existent effects

In this simulation no true effects existed in the experimental landscape ($P(\mathrm{H}_1) = 0$). This situation modeled the pursuit of non-existent effects by an idealized scientific community. Figure S4 shows the results. Because the only significant results from the first

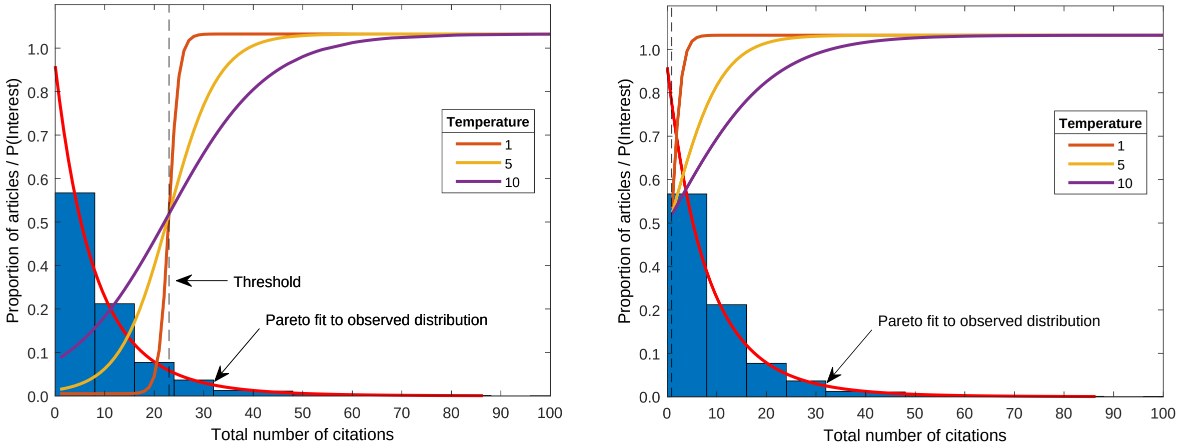

Figure S2. Comparison of different thresholds of interest. The left panel shows the observed distribution of citations for articles published in psychology in 2014 (blue histogram) with a best-fitting pareto distribution (solid red line). The threshold of interest is centered on the 90th percentile of the fitted distribution and is indicated by vertical dashed line. The right panel shows the same citation data but with a threshold of interest on the 10th percentile. The three logistic functions in each panel are centered on the threshold but have different temperatures (see legend). They determine the probability of the scientific community finding a phenomenon to be of interest.

round of experiments reflected Type I errors, the overall cost of experimentation is lower under this perturbation than the conventional situation. Nonetheless, the cost advantage of the public regime persisted. No true interesting effects could be discovered, and the negligible number of falsely identified interesting effects (i.e., a Type I error that was followed by another Type I error) were identical between regimes.

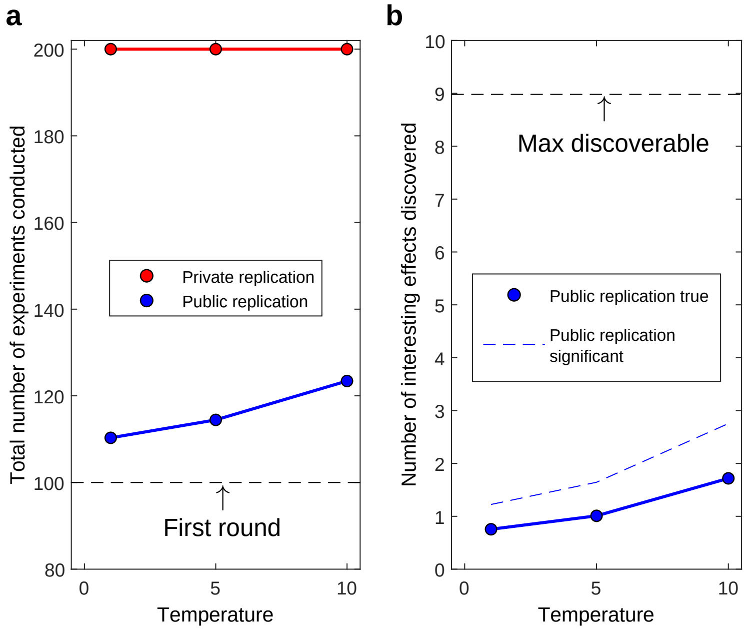

Figure S3. Effects of fraud on discovery-oriented research. All effects are arbitrarily declared significant during the first round. Private replications therefore involve the full set of studies from the first round. Panel **a** shows the total number of experiments conducted. Panel **b** shows the knowledge (true effects discovered) generated by (honest) public replications. Successful replications are identified by the dashed line, and successful replications that are also true (i.e., the null hypothesis was actually false) are identified by plotting symbols and a solid line. Examining private replications for discovered knowledge makes little sense in the fraud scenario, and they are therefore omitted from panel **b**. Temperature refers to the temperature of the logistic decision function (with a threshold at the 90th percentile of citations).

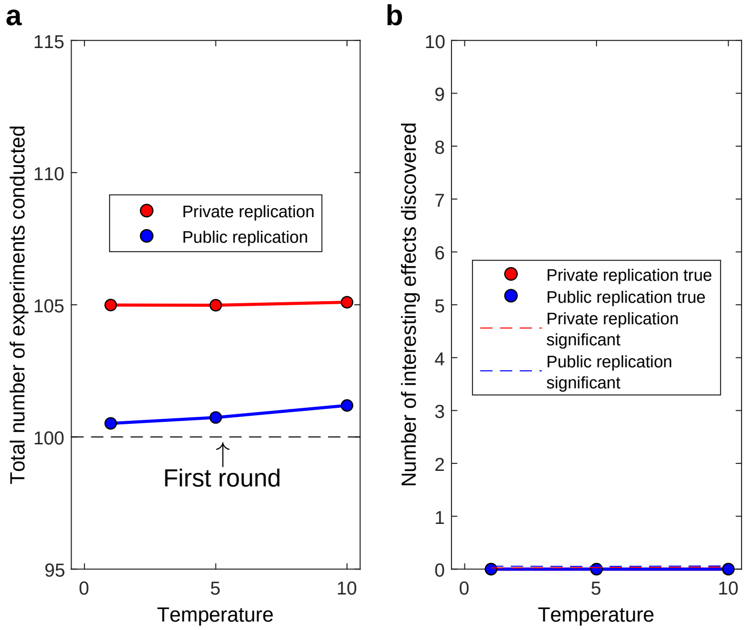

Figure S4. Results when no effects exist in the experimental landscape of ground truths. Panel **a** shows the total number of experiments conducted. Panel **b** shows the number of interesting effects discovered (none of which can be true). Temperature refers to the temperature of the logistic decision function (with a threshold at the 90th percentile of citations).
